# Routine surveillance of patients post Fontan palliation: lessons learnt from cardiac catheterisation

**DOI:** 10.1186/s40949-021-00057-z

**Published:** 2021-03-24

**Authors:** Maria Victoria Ordonez, Giovanni Biglino, Radwa Bedair

**Affiliations:** 1 Bristol Medical School, University of Bristol, Bristol, UK; 2 Adult Congenital Heart Disease Service, University Hospitals Bristol, NHS Foundation Trust, Bristol, UK; 3 National Heart and Lung Institute, Imperial College London, London, UK

**Keywords:** Fontan palliation, Routine diagnostic catheterisation, Clinical indicated catheterisation, Long term follow up

## Abstract

**Background:**

There is no consensus on the clinical utility of ‘routine’ diagnostic cardiac catheterisation in patients with Fontan palliation in the absence of symptoms or haemodynamic lesions.

**Objective:**

We sought to evaluate whether diagnostic cardiac catheterisation for a variety of indications led to a change in the clinical management of patients with a Fontan circulation.

**Methods:**

All adult patients (≥16 years) with Fontan palliation undergoing diagnostic cardiac catheterisation at our institution from 2016 to 2019 were included retrospectively. Patients undergoing electrophysiological studies were excluded as haemodynamic measurements were not taken. Routine cardiac catheterisation at our institution is considered in adult patients who have not had a diagnostic cardiac catheter for more than 5 years.

**Results:**

Thirty-eight patients, mean age 27 ± 7 years, 60% NYHA I, 31% NYHA II, 8% NYHA III, at mean duration post Fontan of 20 ± 6 years, lateral tunnel (LT) *n* = 20, extracardiac (EC) *n* = 14 and atriopulmonary (AP) *n* = 4, underwent 41 diagnostic cardiac catheterisation procedures. Indication for cardiac catheterisation was as follows: haemodynamic lesion identified on cross-sectional imaging in 12; routine catheterisation in 9; cyanosis in 8; dyspnoea in 8; significant liver stiffness on ultrasound hepatic elastography in 2; and arrhythmia in 2. Of the 9 patients undergoing routine diagnostic catheterisation, 3 had not had any diagnostic catheterisation since their Fontan completion and, in the remaining six, the mean time lapsed since the last diagnostic catheter was 8 ± 3 years. The diagnostic catheterisation led to a recommended change in clinical management on 24 occasions (59%): catheter intervention in 17 (40%); surgery in 4 (10%); medication change in 3 (17%); and transplant referral in 2 (5%). The clinical indications that led to changes in clinical management were: cyanosis (8/8), dyspnoea (7/8), haemodynamic lesions on cross-sectional imaging (8/11) and arrhythmia (1/2). None of the 9 patients listed for routine diagnostic catheterisation or as a result of findings on ultrasound hepatic elastography had a recommended change in clinical management.

**Conclusion:**

Diagnostic cardiac catheterisation frequently leads to changes in the clinical management of patients with Fontan palliation presenting with dyspnoea, cyanosis, and for further evaluation of potential haemodynamic lesions identified on cross-sectional imaging. Routine cardiac catheterisation in the absence of the above indications had limited impact on clinical management in our cohort.

## Introduction

Most Fontan patients reach adulthood [1–3]; however, a standardised long-term follow-up strategy has not been agreed, partly reflecting the non-standardised management of these challenging patients across institutions. Fontan patients can develop several complications such as atrial arrhythmias, heart failure, protein losing enteropathy, plastic bronchitis, progressive cyanosis due to veno-venous collateral formation, and Fontan-associated liver disease (FALD), which are the mainstay of Fontan morbidity and mortality [3, 4] (Table 1). The risk and severity of these complications increases over time and with advancing age, and early identification and treatment of any haemodynamic drivers for the development of these complications is essential [3]. Surveillance protocols differ substantially across centres and the unification of these patients’ follow-up remains challenging. Previous studies suggested that these patients present a bimodal pattern, with a cumulative risk of death from thromboembolism increasing during the first 3 years after Fontan palliation and again 15 years after surgery [4, 5], reiterating the importance of surveillance. Recent findings indicate that the use of cardiac MRI for post-Fontan surveillance follows this bimodal trend [1], with significantly greater odds of a change in management following clinically indicated MRI after Fontan as compared to screening MRI. We here present complementary observations based on cardiac catheterisation.

## Methods

**Fig. 1:**
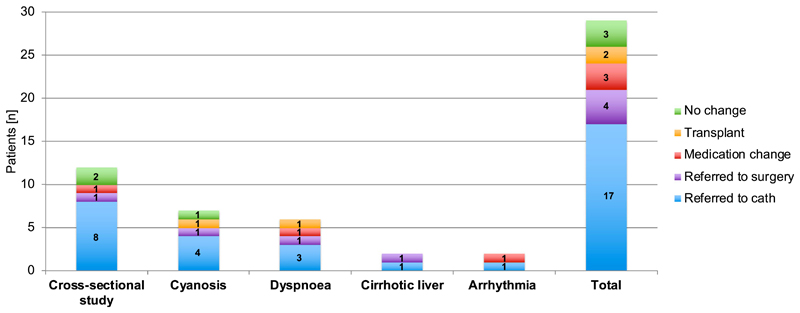
Clinical indications and changes in clinical management post diagnostic catheterisation

We retrospectively analysed *n* = 38 adult Fontan patients who underwent diagnostic cardiac catheterisation between 2016 and 2019, mean follow up post-Fontan palliation: 20 ± 6 years, type of Fontan: LT *n* = 20, EC *n* = 14 and AP *n* = 4. We compared patients who underwent routine diagnostic cardiac catheterization (*n* = 9) versus clinically-indicated diagnostic catheterisation (*n* = 29). Routine catheterisation at our institution is considered in adult patients who have not had a diagnostic cardiac catheter for more than 5 years. Clinical indications for diagnostic catheterisation are displayed in Fig. 1 and included cross-sectional imaging study, arrhythmias, cyanosis, dyspnoea and cirrhotic liver.

## Results

Diagnostic catheterisation led to a recommended change in clinical management in 24 occasions (59%): catheter intervention in 17 (40%); surgery in 4 (10%); medication change in 3 (17%); and transplant referral in 2 (5%) (Fig. 1). None of the 9 patients listed for routine diagnostic catheterisation had a recommended change in clinical management following the procedure.

## Discussion

Similar to MRI observations [1], our findings show limited clinical utility and impact on changing management from routine testing in Fontan patients. However, no definitive conclusion could be drawn based on the small sample. Additionally, the reassurance that a favourable result from a diagnostic cardiac catheterisation study brings to the patient and medical team has not been explored, and may be important. Monitoring for complications in Fontan patients encompasses liver surveillance, single ventricle function assessment, and arrhythmias follow-up. Based on AHA guidelines [3], Fontan patients should be evaluated annually with either echocardiography or cardiac MRI (class I level of evidence C) and cardiac catheterization should be performed in adults before initial Fontan surgery or revision of a prior Fontan connection to assess suitability of pre-intervention hemodynamics for Fontan physiology (class I level of evidence C), in the context of a new onset or worsening atrial tachyarrhythmias (class I level of evidence C), or when the patient is symptomatic and non-invasive testing is insufficient to guide therapy (class IIa level of evidence C), as well as before cardiac transplantation. Evidence-based treatment strategies and preventive management schema are limited, with a need for more widespread consensus as to how best serially monitoring and managing these patients [6] in order to establish a safe protocol.

## Conclusion

Clinically-indicated diagnostic catheterisation frequently leads to changes in the clinical management with Fontan palliation with limited impact on routine diagnostic catheterisation in our cohort. Nonetheless, given the fragile and delicate balance of the univentricular physiology, diagnostic catheterisation might play an essential role as part of the long term follow up in Fontan patients by identifying hemodynamic abnormalities in the context of subtle or even in the absence of clinical manifestations. Therefore, further analysis is warranted.

## Figures and Tables

**Table 1:** Fontan complications and imaging modalities for assessing and following Fontan patients

Fontan Complications	Echo	CMR	CT	Cath	Additional imaging modalities
Conduit Obstruction	**✓✓✓**	**✓✓✓**	**✓✓✓**	**✓✓✓**	
Pulmonary branches stenosis		**✓✓✓**	**✓✓✓**	**✓✓✓**	
Veno-venous collaterals		**✓✓✓**		**✓✓✓**	
Arrhythmias	**✓✓**	**✓✓✓**		**✓✓✓**	
AV valve regurgitation	**✓✓**	**✓✓✓**			
Aortic regurgitation	**✓✓**	**✓✓✓**			
Decreased preload	**✓✓**	**✓✓**		**✓✓✓**	
Systolic dysfunction	**✓✓**	**✓✓✓**			
Increased afterload	**✓✓✓**	**✓✓✓**		**✓✓✓**	
Liver abnormalities	**✓✓✓**	**✓✓✓**	**✓✓✓**		Liver Ultrasound Fibroscan
Protein losing enteropathy	**✓✓✓**	**✓✓✓**		**✓✓✓**	
Plastic bronchitis		**✓✓✓**		**✓✓✓**	Bronchoscopy

## Data Availability

RedCup dataset, Bristol Heart Institute and Bristol University.

## References

[1] Zaki NC, Kelleman MS, James Parks W, Slesnick TC, McConnell ME, Oster ME (2019). The utility of cardiac magnetic resonance imaging in post-Fontan surveillance. Congenit Heart Dis.

[2] Stout KK, Daniels CJ, Aboulhosn JA, Bozkurt B, Broberg CS, Colman JM (2019). 2018 AHA/ACC guideline for the management of adults with congenital heart disease: a report of the American College of Cardiology/American Heart Association task force on clinical practice guidelines. Circulation.

[3] Inglessis I, Landzberg MJ (2007). Interventional catheterization in adult congenital heart disease. Circulation.

[4] Khairy P, Fernandes SM, Mayer JE, Triedman JK, Walsh EP, Lock JE, Landzberg MJ (2008). Long-term survival, modes of death, and predictors of mortality in patients with Fontan surgery. Circulation.

[5] d’Udekem Y, Iyengar AJ, Galati JC, Forsdick V, Weintraub RG, Wheaton GR (2014). Redefining expectations of long-term survival after the Fontan procedure: twenty-five years of follow-up from the entire population of Australia and New Zealand. Circulation.

[6] Rychik J, Atz AM, Celermajer DS, Deal BJ, Gatzoulis MA, Gewillig MH, Hsia TY, Hsu DT, Kovacs AH, McCrindle BW, Newburger JW (2019). Evaluation and management of the child and adult with Fontan circulation: a scientific statement from the American Heart Association. Circulation.

